# Wheat-Meal Bread as a Means of Diminishing Tubercular Disease

**Published:** 1883-01

**Authors:** M Yates

**Affiliations:** Bread Reform League, London


### Wheat-Meal Bread as a means of Diminishing Tubercular Disease.

[By M Yates, Hon. Sec. Bread Reform League, London.]

It is well recognized that defective mineral nutrition is an important factor in the production of rickets and bad teeth, but as its influence in predisposing toward tuberculous disease is not so clearly ascertained, will you kindly allow public attention to be directed to a statement which was discussed at the Social Science and Sanitary Congresses, and which, if confirmed by further scientific research, indicates a simple means of diminishing consumption, which, as Dr. William Farr, F. R. S., says, "is the greatest, the most constant, and the most dreadful of all the diseases that affect mankind." In "Phosphates in Nutrition," by Mr. M. F. Anderson, it is stated that although the external appearances and general condition of a body when death has occurred from starvation are very similar to those presented in tuberculous disease, in starvation, "from wasting of the tissues, caused by the combustion of their organic matter, there would be an apparent *increase* in the percentage proportion of mineral matter; on the other hand, in tubercular disease, there would be a material *decrease* in the mineral matter as compared with the general wasting." Analyses, made by Mr. Anderson, of the vascular tissues of patients who have died of consumption, scrofula, and allied diseases, show "a very marked deficiency in the quantity of inorganic matter entering into their composition; this deficiency is not confined to the organs or tissues which are apparently the seat of the disease, but in a greater or lesser degree pervades the whole capillary system."

The observations of Dr. Marcet, F. R. S., show that in phthisis there is a considerable reduction of the normal amount of phosphoric acid in the pulmonary tissues; and it is very probable that there is a general drain of phosphoric acid from the system.

This loss may be caused by the expectoration and night-sweats, or it may also be produced by defective mineral nutrition, either from deficient supply in the food, or from non-assimilation. But, whatever causes this deficiency, it is universally acknowledged that it is essential the food should contain a proper supply of the mineral elements. If the body is well nourished, the resisting force of the system is raised. Professor Koch and others, who accept the germ theory of disease to its fullest extent, state that the minute organisms of tubercular disease do not occur in the tissues of healthy bodies, and that when introduced into a living body their propagation and increase are greatly favored by a low state of the general health.

Dr. Pavy, F. R. S., showed in his address on the "Dietetics of Bread" that in white flour, instead of obtaining the 23 parts of mineral matter to 100 parts of nitrogenous matter—which is the accepted ratio of a standard diet—we should only get 4.20 parts of mineral matter. Professor Church states that 1 ℔ of white flour has only 49 grains of mineral matter, while 1 ℔ of whole wheat meal has 119 grains. Whole wheat meal, besides containing other essential mineral elements, has double the amount of lime, and nearly three times the amount of phosphoric acid; so that if defective mineral nutrition in any way predisposes to consumption, the adoption of wheat meal prepared in a digestible and palatable form is of primary importance for those who are unable to obtain the phosphates from high-priced animal foods.

Wheat meal has also great advantages for those who are able to afford animal food, for, as Dr. Pavy stated, "It acts as a greater stimulant to the digestive organs."

It is probably due to this stimulating property of wheat meal that people who have adopted it find they can digest animal fat much better than previously. If this is corroborated by general experience, it may be of great benefit in aiding the system to resist tendencies toward consumption and scrofula, for these diseases are generally accompanied by loss of the power of assimilating fat. It is believed that a deficiency of oleaginous matter is a predisposing cause of tuberculous disease. An important prophylactic, therefore, against such maladies, would be a general increase in the use of butter and other fatty foods.

There is such good reason to believe that a low state of nutrition favors the development of tuberculous disease, that parents cannot be too strongly urged to provide their children with a proper supply of healthy, nourishing, and pure food (under which term must, of course, be included pure air and pure water), for by so doing they may often arrest consumptive tendencies, and thus would be diminished the ravages of that fatal disease which, when once established, is "the despair of the physician, and the terror of the public."

